# Comparison between 3-dimensional and 2-dimensional endoscopic thyroidectomy for benign and malignant lesions: a meta-analysis

**DOI:** 10.1186/s12957-021-02134-4

**Published:** 2021-01-21

**Authors:** Zigao Huang, Haiquan Qin, Jiankun Liao, Linghou Meng, Yongjie Qin, Baojia Li, Hao Lai, Xianwei Mo

**Affiliations:** Guangxi Clinical Research Center for Colorectal Cancer, Guangxi Cancer Hospital and Guangxi Medical University Affiliated Cancer Hospital, Nanning, 530021 Guangxi Autonomous Region China

**Keywords:** 3-Dimensional, 2-Dimensional, Endoscopic thyroidectomy, Meta-analysis

## Abstract

**Background:**

The use of 3-dimensional (3D) endoscopic thyroidectomy (ET) has been increasing, but its feasibility and safety have not been well documented for thyroidectomy. Hence, to systematically investigate the comparative outcomes during 3D-ET and 2-dimensional (2D) ET for benign and malignant lesions, we conducted this meta-analysis.

**Methods:**

Based on the PRISMA guidelines, a systematic database search of the PubMed, Cochrane Library, Web of Science, China National Knowledge Infrastructure (CNKI), and Chinese Wanfang databases was performed. The eligible studies were published in English and Chinese up to October 2020. The major endpoints evaluated were procedure time, blood loss, postoperative drainage, postoperative hospitalization, postoperative complications, total number of lymph node dissections (LNDs), and total cost.

**Results:**

A total of 15 relevant studies including 1190 patients (583 for 3D-ET and 607 for 2D-ET) compared the application of 3D and 2D laparoscopic systems in thyroid surgery, of which 8 were endoscopic benign thyroidectomy (EBT) and 7 were endoscopic malignant thyroidectomy (EMT). Our meta-analysis indicated that 3D-ET generally had advantages over 2D-ET in terms of procedure time (*P* = 0.000), blood loss (*P* = 0.000), postoperative drainage (*P* = 0.000), postoperative complications (*P* = 0.000), and LNDs (*P* = 0.006). However, there were no significant differences between the two systems in terms of total cost (*P* = 0.245) or postoperative hospitalization (*P* = 0.068). Subgroup analysis showed consistency of the overall outcomes in each subset, but a shorter postoperative hospitalization in 3D-EBT was revealed.

**Conclusions:**

Compared to 2D-ET, 3D endoscopic thyroidectomy is an efficient, safe, and reliable method with better depth perception and stereoscopic vision, and an equally satisfactory outcome. More clinical RCTs with long-term follow-up are required to reproduce these promising results.

**Supplementary Information:**

The online version contains supplementary material available at 10.1186/s12957-021-02134-4.

## Introduction

Thyroid disease, which includes benign thyroid nodules and malignant thyroid cancers, is a common clinical disease that is on the rise among young women. Since Hüscher [1] first introduced the minimally invasive technique for thyroid surgery in 1996, traditional 2-dimensional endoscopy has become a widespread technique. Compared with standard open thyroidectomy, laparoscopic surgery greatly shortens the operating time, reduces postoperative pain, and reduces surgical trauma [2]. Another compelling advantage of laparoscopic surgery is the cinematic landscape, which made laparoscopic techniques very popular in the field of surgery in the new century.

Owing to the limitations of true depth perception and stereovision, 2D endoscopy can increase the risk of errors in surgical procedures and difficulties in lymph node dissection. Moreover, novice surgeons hardly benefit from the associated reduced learning curves. With the continuous improvement of surgical and endoscopic techniques, 3D laparoscopy has begun to be applied in clinical practice to overcome these drawbacks in different surgeries [3]. 3D endoscopy has been widely used in hepatobiliary surgery [4], neurosurgery [5], gastrointestinal surgery [6, 7], and benign and malignant thyroid lesions [8, 9], even though many surgeons believe that endoscopic surgery is appropriate for benign thyroid disease. 3D imaging systems are conducive to more refined operations and fit the concept of minimally invasive treatment, thanks to the benefit of the high resolution of the 3D volumetric display system in identifying anatomical structures [10]. Nevertheless, some clinical trials and comparative observational studies have indicated that 3D laparoscopic imaging systems have not been widely adopted, even though they have significant advantages in terms of decreased operative time, reduced surgical error rates, and a shorter learning curve for novice surgeons compared to 2D laparoscopic imaging systems [11–13].

Therefore, to systemically investigate the efficiency, safety, and potential advantages of 3D endoscopic thyroidectomy (ET) vs 2D-ET, a meta-analysis was conducted for thyroidectomy with respect to procedure time, blood loss, postoperative drainage, postoperative hospitalization, postoperative complications, total number of lymph node dissections (LNDs), and total cost.

## Methods

The Preferred Reporting Items for Systematic Reviews and Meta-Analyses (PRISMA) statement guidelines [14] were used to perform this meta-analysis to analyze the operability and potential benefits of 3D laparoscopic thyroid surgery versus 2D laparoscopic thyroid surgery.

### Search strategy

We thoroughly searched the PubMed (http://www.ncbi.nlm.nih.gov/pubmed), Cochrane Library (http://www.cochranelibrary.com), Web of Science (http://www.webofscience.com), China National Knowledge Infrastructure (CNKI; https://www.cnki.net/), and Chinese Wanfang (http://www.wanfangdata.com.cn/index.html) databases with the following keywords: “three-dimensional,” “3D,” “two-dimensional,” “2D,” and “thyroidectomy” (up to October 2020). The complete keyword search strings for relevant databases were as follows: (three-dimensional[MeSH Terms] OR 3D[Title/Abstract] OR 3-D[Title/Abstract]) AND (two-dimensional[MeSH Terms] OR 2D[Title/Abstract] OR 2-D[Title/Abstract]) AND (laparoscopic[Title/Abstract] OR laparoscopy[Title/Abstract]) AND (thyroid cancer[MeSH Terms] OR thyroidectomy[Title/Abstract] OR thyroid nodule[Title/Abstract] OR thyroid[Title/Abstract] OR thyroid mass[Title/Abstract]). In addition, we manually searched and reviewed the relevant studies to avoid any omissions, and the keyword search was only limited to studies published in English or Chinese. Any disagreements between the two investigators were resolved by an independent third investigator when necessary.

### Selection criteria

By carefully reading the titles, abstracts, keywords and, if necessary, the full text of the articles, retrieved articles could only be included in our meta-analysis if they met the following inclusion criteria:

1. Randomized or observational studies that were comparative in nature
2. Studies that compared 3D-ET vs 2D-ET
3. Literature published in English or Chinese, and the related outcomes could be extracted directly or calculated indirectly
4. Two or more of the following results were reported: procedure time, blood loss, postoperative drainage, postoperative hospitalization, postoperative complications, LNDs, and total cost

Exclusion criteria were as follows:

1. Case reports, editorials, review articles, commentary articles, robotic-assisted surgeries and quasi-randomized trials
2. Duplicate data from different articles
3. Transoral endoscopic thyroid surgeries

### Data extraction

Two investigators (ZH and HQ) performed quality assessments of all eligible studies, and disagreements between the two investigators were resolved by an independent third investigator (JL) when necessary. The following data were independently extracted, if available, and summarized in Table 1: the first author, publication year, city/country, study type, sex, mean age, number of participants, diseases/surgical approach, 3D system, surgical outcomes of interest (procedure time, blood loss), postoperative complications, postoperative hospitalization, total cost, and total number of LNDs.

**Table 1:** Demographics of the included studies comparing 2D and 3D ET

First author/year	City/country	Study type	NOS stars/Jadad score	3D VS. 2D						Diseases/surgical approach	3D system
				No. of participants		Gender (male/female, *n*)		Mean age (year)^a^			
Tang Tao, 201 9[15]	Sichuan, China	RS	7★/−	32	36	2/30	1/35	39.25 ± 8.73	41.72 ± 7.94	Thyroid cancer/via breast approach	NR
Chen Jian, 2015 [16]	Hubei, China	RS	8★/−	26	34	6/20	6/28	46.15 ± 11.74	49.29 ± 11.61	Thyroid diseases/via modified chest and mammary areola approach	NR
Liu Xue-Wen, 2017 [17]	Guangdong, China	RS	7★/−	30	30	5/25	4/26	41.40 ± 9.07	40.93 ± 7.65	Thyroid cancer/via breast approach	NR
Huang Jue, 2020 [18]	Henan, China	RCT	−/3	32	32	7/25	10/22	55.27 ± 6.34	56.81 ± 7.10	Thyroid cancer/via breast approach	Olympus Corporation, Japan
Jiang Yinhai, 2019 [19]	Shandong, China	RS	8★/−	40	40	6/34	7/33	41.10 ± 6.80	43.10 ± 7.20	Thyroid cancer/via breast approach	Viking 3D HD system, USA
Feng Jianping, 2020 [20]	Guangdong, China	RS	8★/−	26	25	4/22	2/23	30.27 ± 7.95	31.44 ± 6.97	Thyroid cancer/via breast approach	Viking 3D HD system, USA
Xing Ying, 2018 [21]	Beijing, China	RCT	−/3	42	42	10/32	8/34	47.60 ± 8.50	49.30 ± 9.50	Thyroid cancer/via modified chest and mammary areola approach	NR
Zou Zhaowei, 2014 [22]	Guangdong, China	RS	7★/−	30	30	10/20	12/18	43.30 ± 7.81	44.40 ± 7.59	Benign thyroid nodules/via breast approach	NR
Jiasheng Xu, 2018 [23]	Jiangxi, China	RS	7★/−	88	92	NR	NR	29.51 ± 5.36	34.36 ± 5.53	Benign thyroid nodules/via breast approach	NR
Zi-Fang Zheng, 2018 [24]	Fujian, China	RS	7★/−	50	50	12/38	15/35	36.70 ± 7.50	37.30 ± 7.80	Thyroid diseases/via the trans-thoracoareolar approach	Karl Storoz, Germany
Zhao Bei-yong, 2020 [25]	Henan, China	RCT	−/3	69	69	7/62	9/60	36.85 ± 7.11	36.21 ± 6.97	Benign thyroid nodules/via breast approach	NR
Li Dongwei, 2018 [26]	Guangdong, China	RS	8★/−	26	35	3/23	5/30	34.60 ± 9.00	35.50 ± 9.20	Benign thyroid nodules/via breast approach	Olympus Corporation, Japan
Fan Dunhui, 2019 [27]	Gansu, China	RS	7★/−	35	35	19/16	20/15	41.16 ± 5.02	40.25 ± 4.56	Benign thyroid nodules/via breast approach	Karl Storoz, Germany
Li Weiqi, 2017 [28]	Guangdong, China	RCT	−/3	20	20	5/15	6/14	43.10 ± 7.20	40.70 ± 8.00	Benign thyroid nodules/via breast approach	Karl Storoz, Germany
Zhang Duojun, 2017 [29]	Neimenggu, China	RS	7★/−	37	37	4/33	7/30	36.25 ± 5.28	37.2 ± 7.12	Benign thyroid nodules/via breast approach	Olympus Corporation, Japan

*NOS* Nottingham-Ottawa scale, *3D* 3-dimensional, *2D* 2-dimensional, *RS* retrospective study, *RCT* randomized controlled trial, *NR* not reported, *ET* endoscopic thyroidectomy

^a^Data are the mean ± standard deviation

★ Number of stars for Nottingham-Ottawa scale for each included trial

### Quality assessment of the studies

For retrospective studies, two independent reviewers (ZH and HQ) evaluated the quality assessment of nonrandomized controlled trials using the Newcastle-Ottawa Quality Assessment Scale (NOS; 9 points) [30]. Quality assessment focused on selection, comparability, and the outcomes of each study. Selection and outcome received up to one star for each numbered item, while comparability was given up to two stars. Each study was scored quantitatively according to these established criteria. Studies with 6–9 stars on the quality assessment were classified as high quality, while those with < 5 stars were excluded.

For the quality assessment of randomized clinical trials, we used the Jadad score (5 points) [31] for assessment. Items in this assessment include randomization, double blinding, withdrawals, and dropouts. Studies with 3–5 points were considered high quality and were included; otherwise, they were excluded from our meta-analysis.

### Statistical analysis

This analysis compared the efficacy, safety, and overall clinical outcomes of 3D-ET vs. 2D-ET. STATA V.12.0 A (Stata Corp, College Station, TX, USA) was used to analyze all available data in our study for comparison. Furthermore, the relative risk (RR) and 95% confidence interval (CI) were used to analyze dichotomous variables. The weighted mean difference (WMD) and 95% CI were calculated for six continuous outcomes (procedure time, blood loss, postoperative drainage, postoperative hospitalization, LNDs, and total cost). The random-effects model was used for data analysis when heterogeneity existed between studies; for all others, a fixed-effects model was used [32]. When necessary, subgroup analysis of the study variables was conducted. In addition, sensitivity analysis was performed to assess bias. Funnel plots and Begg’s and Egger’s tests were performed to detect study bias [33]. If *p* was < 0.05, a statistically significant difference was present among the studies (Table 2).

**Table 2:** Outcomes of the meta-analysis comparing 3D and 2D ET

Variables	No. of studies	Patients (3D vs 2D)	Heterogeneity		Model	WMD/RR, (95% CI)	*P* value
			*I*^2^	*P* value			
Procedure time	15	583/607	88.4%	0.000	Random	− 14.95 (− 18.48, − 11.42)	0.000
Blood loss	15	583/607	93.6%	0.000	Random	− 6.91 (− 9.20, − 4.62)	0.000
Postoperative drainage	13	511/535	69.0%	0.000	Random	− 2.98 (− 5.50, − 0.46)	0.000
Postoperative hospitalization	14	557/573	18.4%	0.253	Fixed	− 0.10 (− 0.21, − 0.01)	0.068
Postoperative complications	12	501/525	0.0%	0.959	Fixed	0.56^a^ (0.41, 0.77)	0.000
LNDs	4	128/131	0.0%	0.782	Fixed	0.67 (0.19, 1.15)	0.006
Total cost	8	288/296	85.8%	0.000	Random	275.10 (− 188.57, 738.76)	0.245

*ET* endoscopic thyroidectomy, *3D* 3-dimensional, *2D* 2-dimensional, *LNDs* the total number of lymph node dissections, *WMD/RR* weighted mean difference/relative risk, *CI* confidence interval

^a^Relative risk

## Results

### Study retrieval

**Fig. 1:**
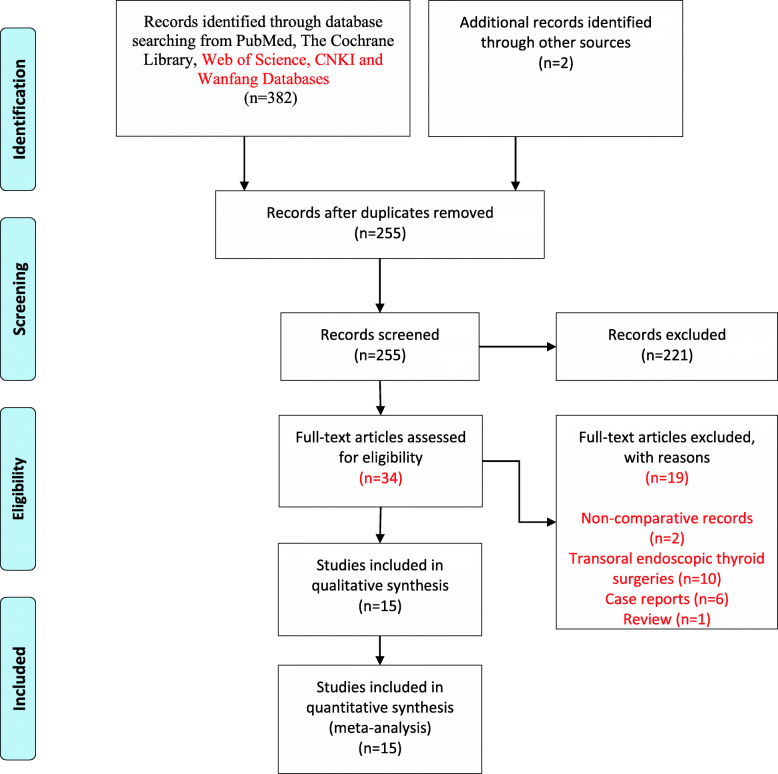
The process of study selection performed: PRISMA flow diagram

Using the described search strategy, a total of 382 potential records were initially identified from the electronic databases, and 2 more records were manually identified from other sources. A total of 255 records remained after duplicate articles were removed. Among these, 221 records were directly eliminated after carefully filtering based on the titles and abstracts of these relevant studies, and the remaining 34 articles were further evaluated. Eventually, searches of the electronic databases generated 15 studies (4 randomized and 11 retrospective) comparing 3D and 2D imaging systems during different thyroid surgeries. The study selection process performed is presented in the PRISMA flowchart (Fig. 1).

### Characteristics of the included studies

Our study included a total of 1190 patients with benign or malignant thyroid lesions, 583 underwent 3-dimensional endoscopic thyroidectomy and 607 underwent conventional 2-dimensional endoscopic thyroidectomy. All of the studies were conducted in different parts of China and published from 2014 to 2020. In addition, of the 15 suitable studies [15–29], eight studies [22–29] involved thyroid nodules and seven studies [15–21] involved thyroid carcinoma tumors. The surgeries in both treatment groups involved different extents of thyroidectomy, such as lobectomy, lobectomy + central neck dissection, and total thyroidectomy +central neck dissection. The 3D systems involved in these studies were mainly from the USA (Viking 3D HD system), Germany (Karl Storoz), and Japan (Olympus Corporation), and some were unknown. The demographics of the included studies comparing 2D-ET and 3D-ET are shown in Table 1.

### Quality assessment of eligible studies

Based on the NOS, two independent reviewers evaluated the quality of each eligible study, and all the included studies scored seven or more stars; therefore, they were considered high quality. Moreover, each included RCT was strictly judged according to the Jadad score. The total scores for each article are presented in Table 1.

### Meta-analysis

#### Study endpoints

**Fig. 2:**
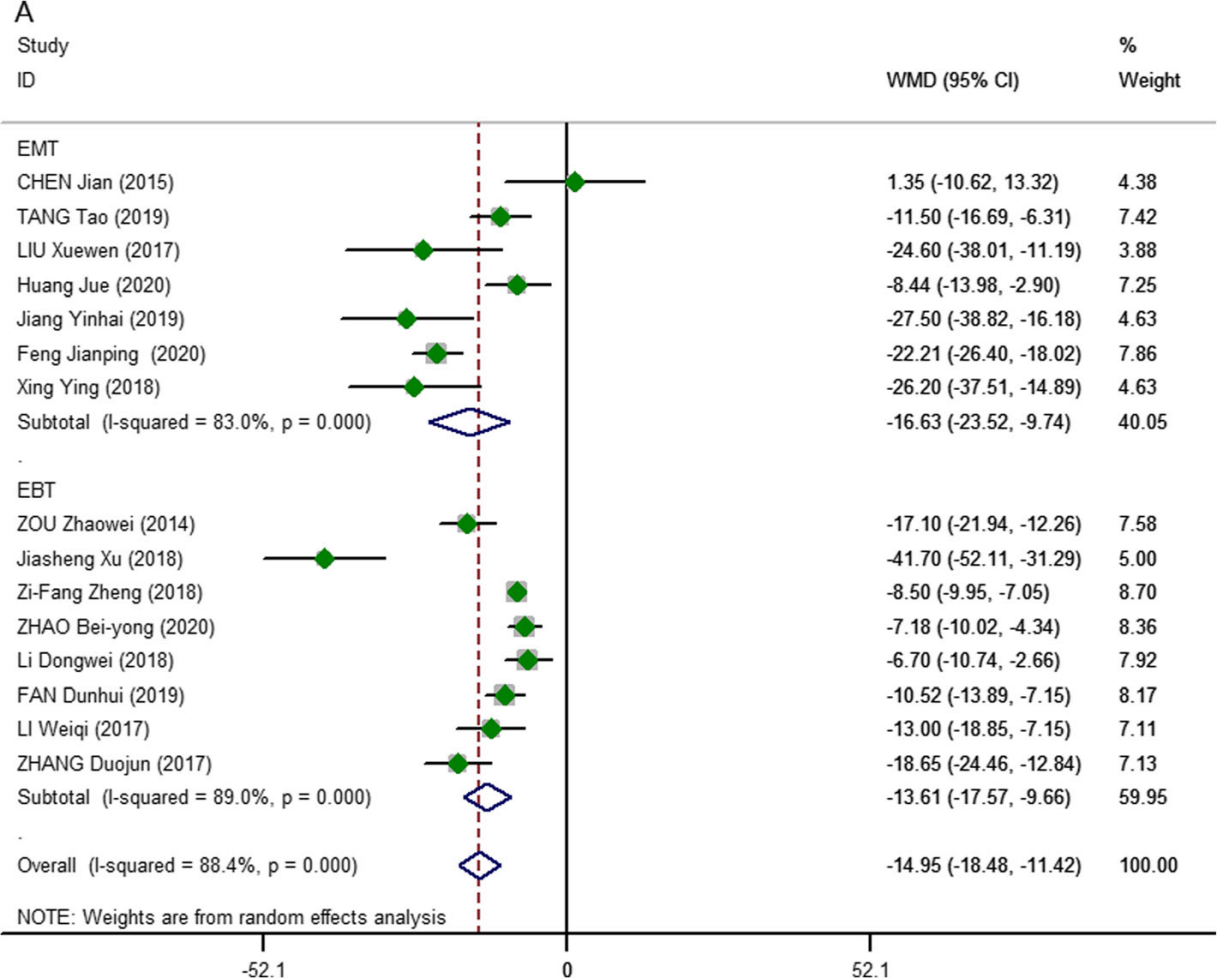
Meta-analysis forest plots of comparisons between 3D-ET and 2D-ET concerning procedure time

Fifteen studies [15–29] on 3D vs 2D endoscopic thyroidectomy (583 vs. 607 patients, respectively) were included in the analysis of procedure time. Meta-analysis demonstrated that 3D endoscopic thyroidectomy was shorter than 2D-ET in terms of procedure time (pooled WMD, − 14.95; 95% CI, − 18.48, − 11.42; *P* = 0.000; *I*^2^ = 88.4%), regardless of whether the procedure was an endoscopic benign thyroidectomy (EBT; WMD, − 13.61; 95% CI, − 17.57, − 9.66; *P* = 0.000; *I*^2^ = 89.0%) or endoscopic malignant thyroidectomy (EMT; WMD, − 16.63; 95% CI, − 23.52, − 9.75; *P* = 0.000; *I*^2^ = 83.0%). Because of the high heterogeneity of the studies, we chose the random-effects model for our meta-analysis (Fig. 2).

**Fig. 3:**
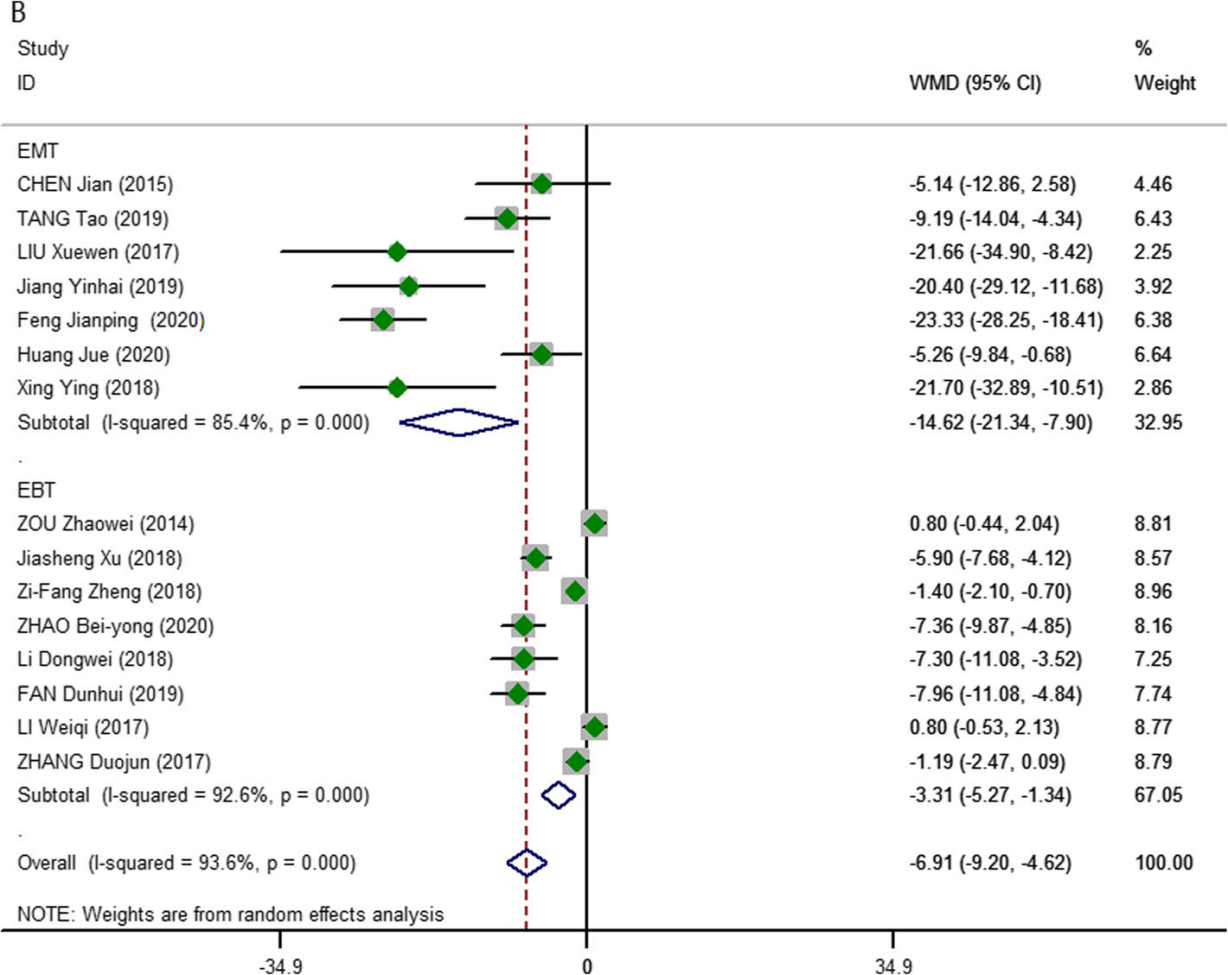
Meta-analysis forest plots of comparisons between 3D-ET and 2D-ET concerning blood loss

Regarding blood loss, 15 studies [15–29] provided available data regarding blood loss volume, and they included 1190 patients. The pooled result showed that the 3D group had significantly less intraoperative blood loss (pooled WMD, − 6.91; 95% CI, − 9.20, − 4.62; *P* = 0.000; *I*^2^ = 93.6%). In addition, the results revealed that the 3D group had less blood loss than the 2D group in the subgroup analysis (EBT: WMD, − 3.31; 95% CI, − 5.27, − 1.34; *P* = 0.000; *I*^2^ = 92.6%; EMT: WMD, − 14.62; 95% CI, − 21.34, − 7.90; *P* = 0.000; *I*^2^ = 85.4%). The above analysis adopted the random-effects model due to high heterogeneity (Fig. 3).

**Fig. 4:**
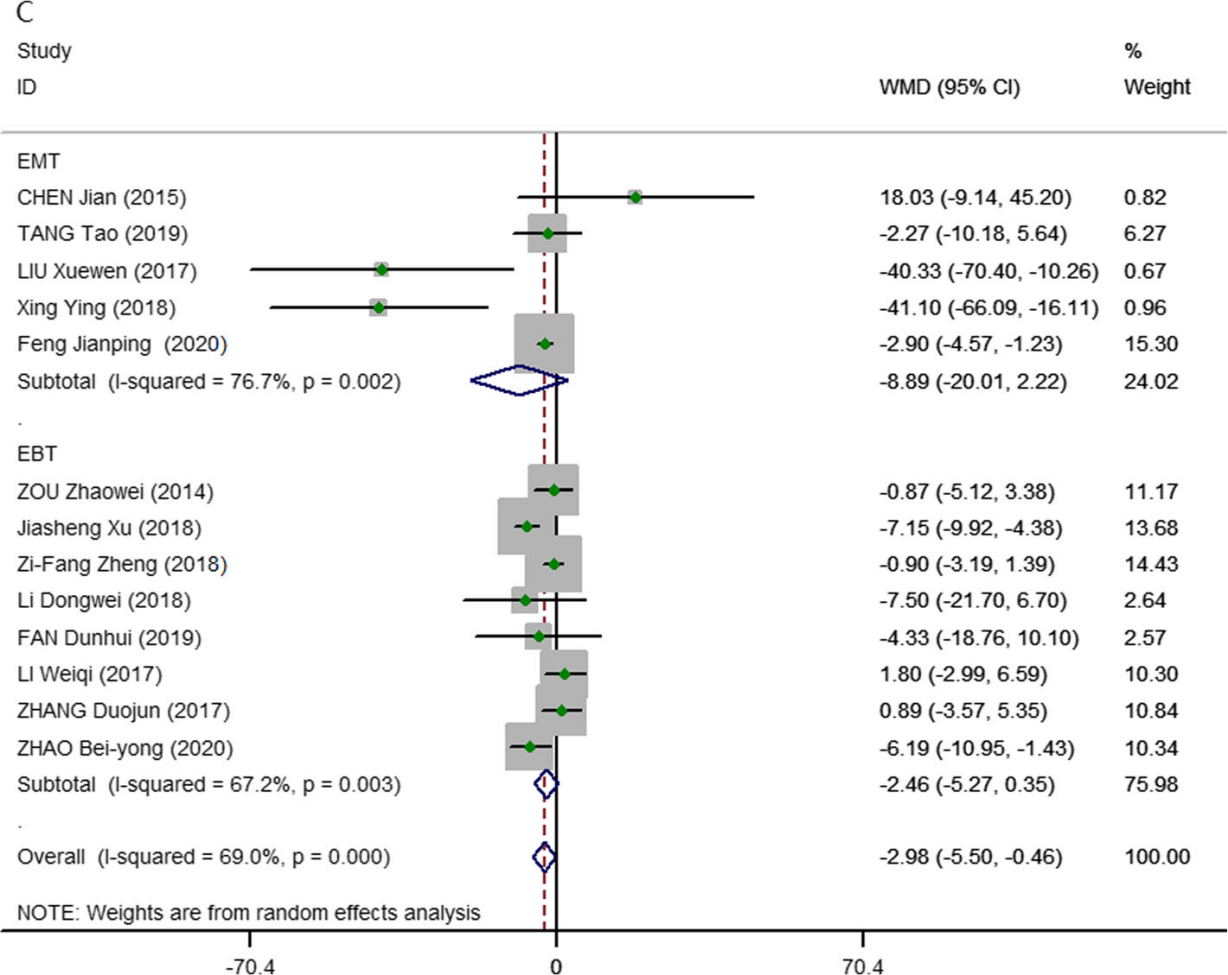
Meta-analysis forest plots of comparisons between 3D-ET and 2D-ET concerning postoperative drainage

All studies [15–29] provided data on postoperative drainage. There were significant differences in postoperative drainage (pooled WMD, − 2.98; 95% CI, − 5.50, − 0.46; *P* = 0.000; *I*^2^ = 69.0%) between the 3D and 2D imaging groups. A random-effects model was used due to the relatively high heterogeneity in our analysis. The same results were found in subgroup analysis between laparoscopic benign thyroidectomy (WMD, − 2.98; 95% CI, − 5.50, − 0.35; *P* = 0.003; *I*^2^ = 67.2%) and laparoscopic malignant thyroidectomy (WMD, − 8.90; 95% CI, − 20.01, − 2.22; *P* = 0.000; *I*^2^ = 76.7%). The heterogeneities were significant, and a random-effects model was used in this analysis (Fig. 4).

**Fig. 5:**
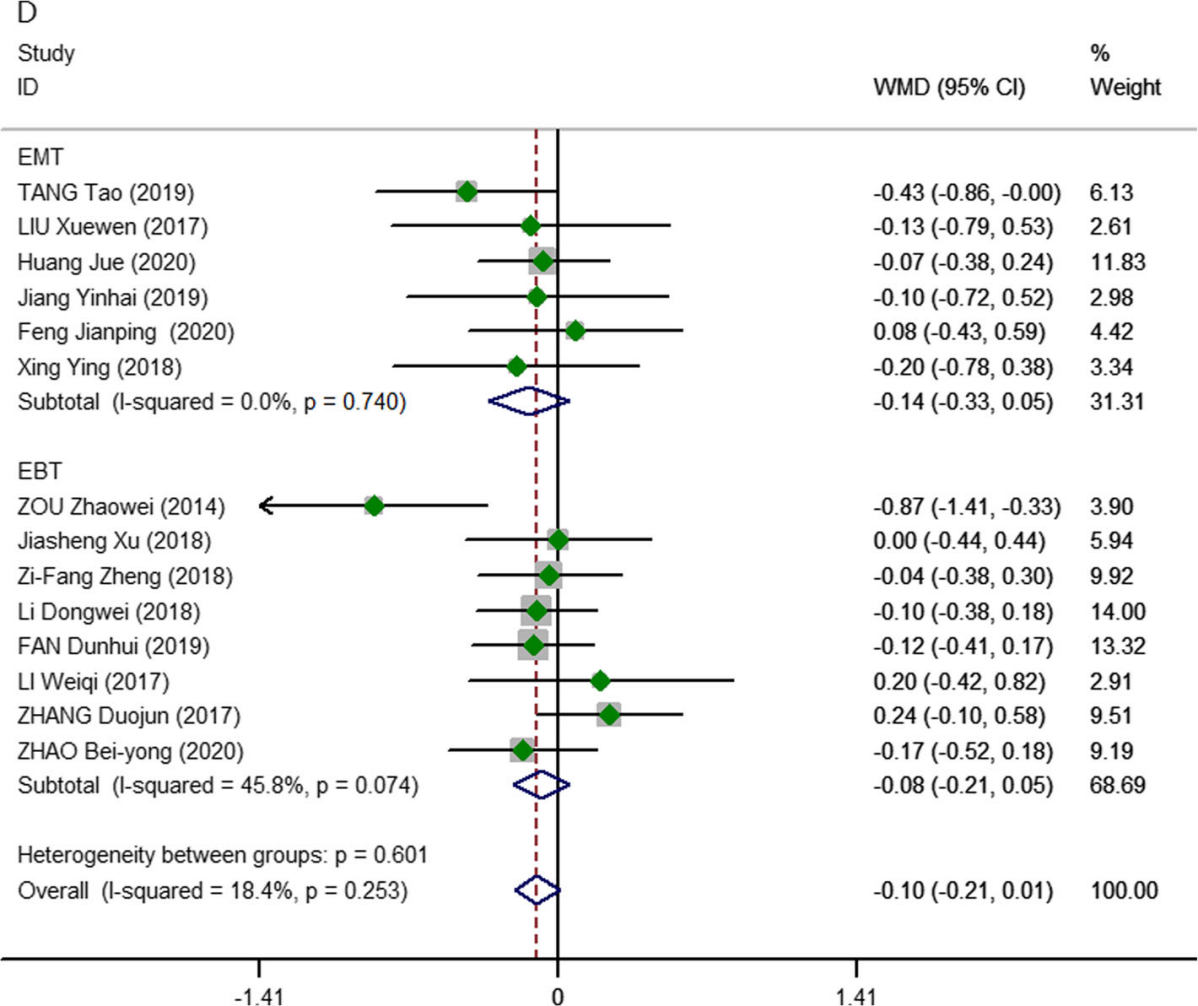
Meta-analysis forest plots of comparisons between 3D-ET and 2D-ET concerning postoperative hospitalization

In our meta-analysis, Chen Jian [16] did not report the length of hospital stay, but the other studies [15, 17–29] that did included 1130 patients (557 vs. 573 patients, respectively). No significant differences were observed between patients who underwent 2D and 3D display procedures (pooled WMD, − 0.10; 95% CI, − 0.21, − 0.01; *P* = 0.068), with relatively low heterogeneity (*I*^2^ = 18.4%). At the same time, we found that 3D endoscopic thyroidectomy was not significantly shorter than 2D in the subgroup analysis of both laparoscopic benign and malignant thyroidectomy (Fig. 5).

**Fig. 6:**
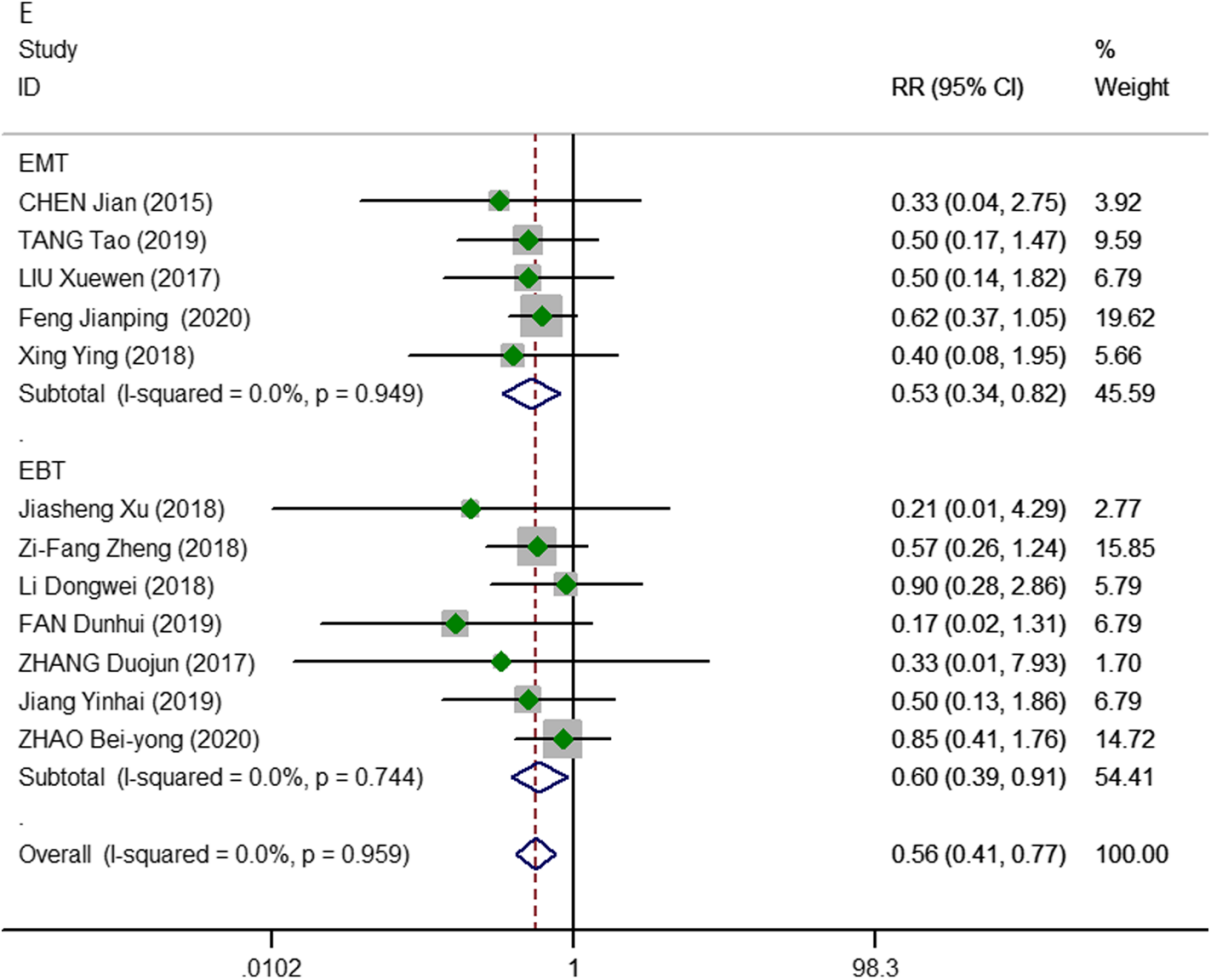
Meta-analysis forest plots of comparisons between 3D-ET and 2D-ET concerning postoperative complications

In terms of postoperative complications, hoarseness, hypocalcemia, subcutaneous congestion, subcutaneous effusion, and cough were mentioned in 12 studies [15–17, 19–21, 23–27, 29] (501 vs. 525 patients, respectively). Compared with the 2D endoscopic thyroidectomy group, the 3D group was observed to be more advantageous in terms of the number of postoperative complications (pooled RR, 0.56; 95% CI, 0.41, 0.77; *P* = 0.000; *I*^2^ = 0.0%). The same results were observed in the subgroup analysis, regardless of whether laparoscopic thyroidectomy for thyroid nodules (RR, 0.60; 95% CI, 0.39, 0.91; *P* = 0.017; *I*^2^ = 0.0%) or malignant thyroid cancers (RR, 0.53; 95% CI, 0.34, 0.82; *P* = 0.004; *I*^2^ = 0.0%) were performed (Fig. 6).

**Fig. 7:**
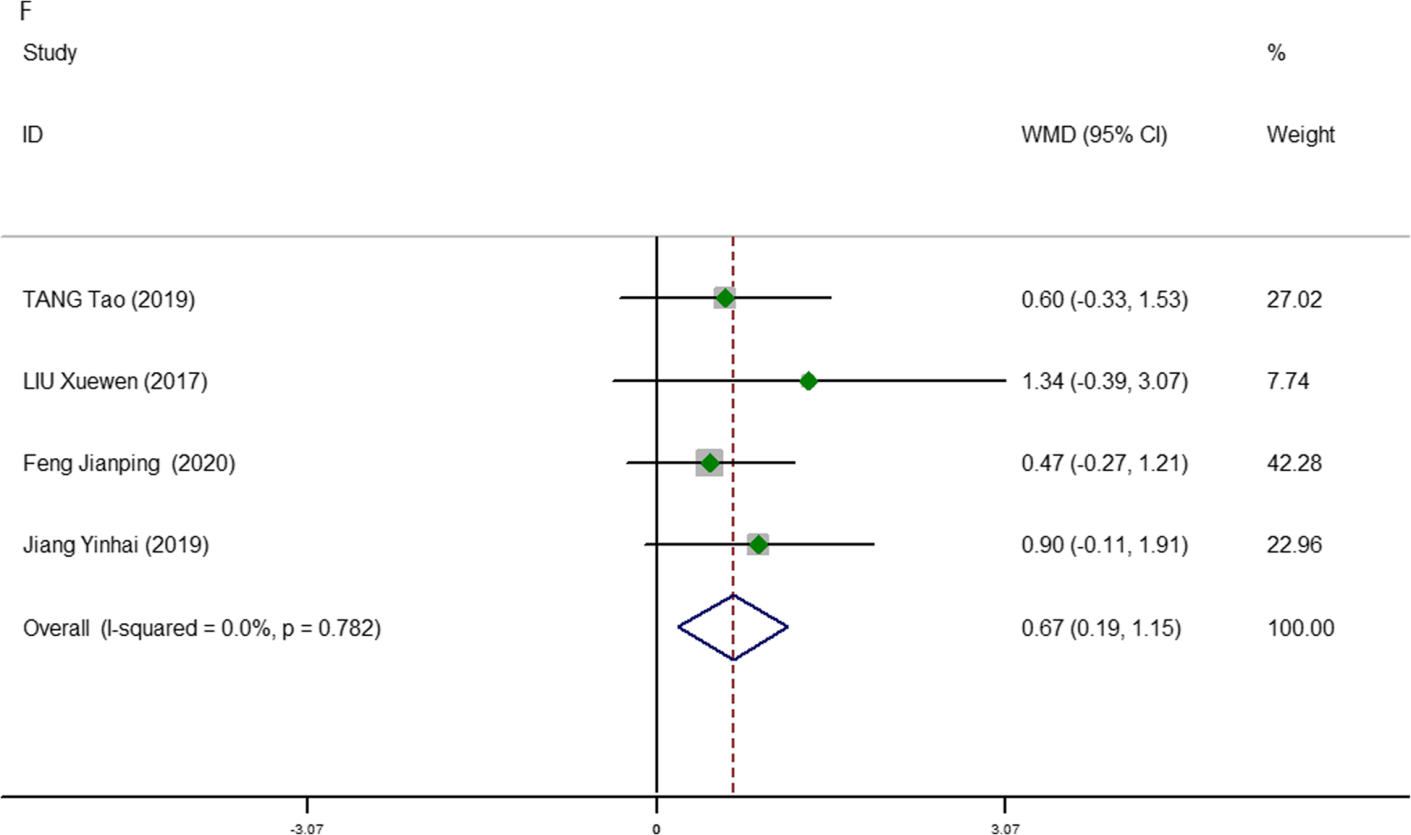
Meta-analysis forest plots of comparisons between 3D-ET and 2D-ET concerning total number of LNDs

Comparison of the total number of LNDs between 3D and 2D endoscopic thyroidectomy was reported in four studies [15, 17, 19, 20]. We found that the number of LNDs were significantly higher in the 3D group than in the 2D group (WMD, 0.67; 95% CI, 0.19, 1.15; *P* = 0.006; *I*^2^ = 0.0%). Additionally, regarding the total number of LNDs, there appeared to be a better advantage in the 3D group, in both EBT and EMT (Fig. 7).

**Fig. 8:**
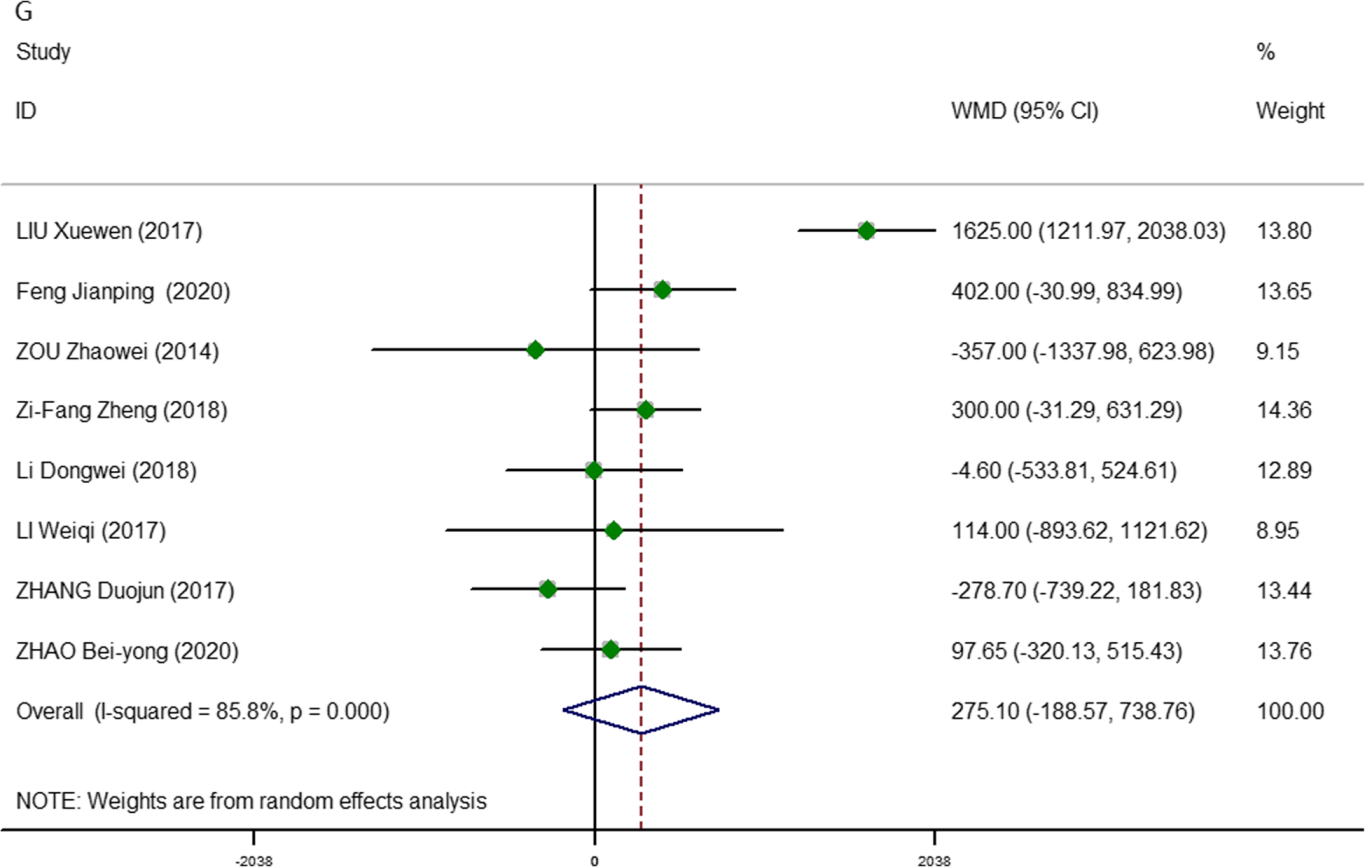
Meta-analysis forest plots of comparisons between 3D-ET and 2D-ET concerning total cost

All data on total cost reported in the included studies have been compiled and presented in Fig. 8 [17, 20, 22, 24–26, 28, 29]. The random-effects model was applied to analyze the total hospital expenses. The results contrasted markedly, and the meta-analysis failed to show a benefit of 3D of the same magnitude as that documented in other 2D models in patients with thyroid problems (pooled WMD, 275.10; 95% CI, − 188.57, 738.76; *P* = 0.245). However, the heterogeneity among studies was moderately high (*I*^2^ = 85.8%).

#### Sensitivity analysis and publication bias

In our study, we performed sensitivity analysis by eliminating each study in turn to investigate the impact of each study on the overall summary estimates. Our meta-analysis results showed that the impact of each individual dataset on the overall estimates was not statistically significant. Publication bias was analyzed in terms of our treatment outcomes; there was no publication bias, as funnel plots and Begg’s and Egger’s test results indicated.

## Discussion

In 2D imaging systems, surgeons need to accumulate experience and constantly improve micromanipulation techniques to overcome operational errors due to a lack of depth perception and stereovision. Compared with surgery for benign diseases, more complex thyroid cancer surgeries require advanced laparoscopic techniques, such as intracorporeal suturing and knotting [34]. It is widely believed that the emergence of 3D laparoscopic surgery is another milestone in surgery and changes the status of traditional laparoscopic surgery in the treatment of various diseases, especially with more complex surgeries, such as deep lymph node dissection and intestinal anastomosis. In several related studies, the 3D endoscopy-assisted system successfully showed a better outcome than the traditional 2D system [13, 35, 36]. 3D imaging systems optimize picture quality and visual experience, which helps surgeons improve surgical skills in a short time and shortens the learning curve [11, 37]. Aside from the conventional three-dimensional imaging system, 3D robotic-assisted surgery and glass-free 3D endoscopic systems have gradually been applied in clinical practice and show some advantages [38, 39]. Unfortunately, the new generation of 3D laparoscopy, which has received much attention, has not been widely adopted due to its high purchase price. Thus, the traditional 3D endoscopic display may represent the best choice for many centers.

In this meta-analysis, we compared surgical outcomes of interest (procedure time, blood loss) and short-term efficacy between 3D and 2D endoscopic thyroidectomy for benign and malignant lesions. Overall, our meta-analysis results indicated that 3D endoscopic thyroidectomy has successfully shown numerous relative advantages over 2D-ET, such as procedure time, intraoperative blood loss, postoperative drainage, postoperative complications, and number of LNDs. In addition, we have no firm evidence to indicate that 3D systems lead to an increase in the total cost and length of postoperative hospitalization, even though there was an advantage in terms of the length of hospital stay for benign 3D endoscopy thyroidectomy.

In terms of the surgical time and blood loss volume during surgery, our meta-analysis indicated that the 3D display system played a more effective role in endoscopic thyroidectomy. The camera system of the 3D endoscope is composed of two separate cameras, which combine slightly different viewpoints to produce stereo vision. Although older versions of the 3D display system have caused surgeon discomfort to some extent, such as dizziness and blurred vision [40], 3D laparoscopic surgery provides better picture quality and stereoscopic vision and greatly minimizes these disadvantages. Moreover, visualization of three-dimensional laparoscopy is helpful to estimate anatomical depth and improve the accuracy of surgical operations [41], which explains the decreased operative time and reduced blood loss of 3D endoscopic-assisted surgery. Our results were the same as those of Fergo [12] and Xue-Wen Liu [17]; however, Jun Lu [13] argued that, while 3D laparoscopic surgery reduced blood loss, there was no significant difference in operation time between the 3D and 2D groups. Several comparative studies [16, 42] showed no statistically significant difference between the duration of surgery and intraoperative bleeding. Therefore, further studies may be required to confirm the surgical outcome of 3D endoscopic thyroidectomy.

The harvested lymph node ratio was an independent predictor of regional lymph node recurrence in patients with papillary thyroid carcinoma [43]. Interestingly, our results showed that compared with 2D endoscopic displays, 3D endoscopic displays have advantages in terms of LNDs. Feng et al. [44] also reported that 3D stereoscopic imaging reduced both the duration of lymph node dissection and the overall operative time during laparoscopic radical cystectomy with pelvic lymph node dissection. 3D laparoscopic visualization and the associated better image quality play a decisive role in distinguishing anatomical structures, which may be beneficial for dissecting lymph nodes and more complicated surgeries.

Furthermore, we found no evidence to support a higher hospitalization expenses associated with 3D imaging systems in our pooled analysis, even though the 3D group was associated with a slightly higher but acceptable length of hospitalization for laparoscopic benign thyroidectomy. Generally, the total cost of hospitalization is associated with the postoperative hospitalization length of stay, which also showed no statistically significant difference in our results. It is obvious that the length of stay is variable and mainly relies on the doctor’s subjective assessment; thus, it is not expected that 3D endoscopic display has a similar total cost and postoperative hospitalization as 2D. There were no significant differences in the amount of postoperative drainage between the two groups.

Regarding postoperative complications and overall complication incidence, our meta-analysis showed that 3D-ET was superior to 2D-ET in both laparoscopic benign thyroidectomy and laparoscopic malignant thyroidectomy. We acknowledged that there were varying quality standards for reporting complications in the included studies, even though the heterogeneity of postoperative complications in the included studies was low, indicating that our findings regarding these outcomes were reliable.

Admittedly, numerous limitations still exist in our meta-analysis. First, of the studies we included, only four were RCTs, and the others were retrospective studies. Additionally, it is difficult to acquire unpublished data, which increases the risk of selection and publication bias. In addition, all of the studies were from China, potentially limiting the applicability of the clinical effectiveness findings to patients of Chinese descent. Moreover, heterogeneity in some of the results still existed because of the differences in patient selection, surgical equipment, levels of surgeon experience, and surgical approaches. Last but not the least, we did not take into account the long-term outcome of 3-D endoscopic thyroid surgery. However, we could still draw some conclusions after considering the limitations.

## Conclusions

Overall, the 3D endoscopic system was superior to the 2D endoscopic system in terms of procedure time, blood loss, postoperative drainage, postoperative complications, and number of LNDs. Nevertheless, 3D-ET had no advantages for total cost or postoperative hospitalization. More clinical RCTs with long-term follow-up are required to reproduce these promising results.

## Supplementary Information

**Additional file 1.**

**Additional file 2.**

## Data Availability

All data generated or analyzed during this study are included in this published article.

## Abbreviations

3D: 3-Dimensional
2D: 2-Dimensional
ET: Endoscopic thyroidectomy
LNDs: Lymph node dissections
EBT: Endoscopic benign thyroidectomy
EMT: Endoscopic malignant thyroidectomy
RCTs: Randomized controlled trials
PRISMA: Preferred Reporting Items for Systematic Reviews and Meta-Analyses
CNKI: China National Knowledge Infrastructure
MeSH: Medical subject heading
NOS: Newcastle-Ottawa Quality Assessment Scale
RR: Relative risk
CI: Confidence interval
WMD: Weighted mean difference
