# Supplementary material for: Comparison between 3-dimensional and 2-dimensional endoscopic thyroidectomy for benign and malignant lesions: a meta-analysis
Source: World J Surg Oncol. 2021 Jan 21;19:23. doi: 10.1186/s12957-021-02134-4 (PMC7819346; doi:10.1186/s12957-021-02134-4)
Supplement: Supplementary file 1 — Additional file 1. [file 12957_2021_2134_MOESM1_ESM.docx]

Dear readers,

We now show the detailed replicable search strategy for each database in our manuscript ID WJSO-D-20-01756 entitled “Comparison between 3-Dimensional and 2-Dimensional Endoscopic Thyroidectomy for Benign and Malignant Lesions: A Meta-Analysis” to you. There were duplicate results between the following single database search results.

The complete keyword search strings for the Pubmed (http://www.ncbi.nlm.nih.gov/pubmed) were: (three-dimensional[MeSH Terms] OR 3-dimensional[Title/Abstract] OR 3D[Title/Abstract] OR 3-D[Title/Abstract]) AND (laparoscop*[Title/Abstract] OR endoscop*[Title/Abstract]) AND (thyroid*[Title/Abstract]). The process of study selection was as follows:

Additional records identified through other sources
(n=0)

Records identified through database searching from PubMed (n=30)

Records after duplicates removed
(n=30)

Records excluded
(n=16)

Records screened (n=30)

Full-text articles excluded, with reasons
(n=7)

Transoral endoscopic thyroid surgeries (n=3)

Case reports (n=4)

Full-text articles assessed for eligibility (n=14)

Studies included in quantitative synthesis (meta-analysis)
(n=7)

The keyword search strings for The Cochrane Library (<http://www.cochranelibrary.com>) were: (3D OR 3-D OR 3-dimensional OR three-dimensional) AND thyroid* in Title Abstract Keyword. Unfortunately, we did not retrieve relevant studies in this database.

The retrieval formula of Web of Science (http://www.webofscience.com) were as follows: TS=(3D OR 3-D OR 3-dimensional OR three-dimensional) AND TS=(laparoscop* OR endoscop*) AND TS=thyroid*. The process of study selection was as follows:

Additional records identified through other sources
(n=0)

Records identified through database searching from Web of Science (n=36)

Records after duplicates removed
(n=36)

Records excluded
(n=26)

Records screened (n=36)

Full-text articles excluded, with reasons
(n=8)

Transoral endoscopic thyroid surgeries (n=4)

Case reports (n=3)

Review (n=1)

Full-text articles assessed for eligibility (n=10)

Studies included in quantitative synthesis (meta-analysis)
(n=2)

The English search terms for the CNKI (<https://www.cnki.net/>) and Chinese Wanfang (<http://www.wanfangdata.com.cn/index.html>) were: (3D OR 3-D OR 3-dimensional OR three-dimensional) AND (thyroid OR thyroidectomy) AND (laparoscop* OR endoscop*). Besides, the Chinese search terms for the CNKI (<https://www.cnki.net/>) and Chinese Wanfang (<http://www.wanfangdata.com.cn/index.html>) were: (3D OR 3维 OR 三维) AND (甲状腺 OR 甲状腺切除术) AND (腔镜 OR 内镜). The process of study selection was as follows:

Full-text articles assessed for eligibility (n=29)

Records screened (n=189)

Additional records identified through other sources
(n=2)

Records identified through database searching from CNKI AND Chinese Wanfang (n=316)

Records excluded
(n=160)

Full-text articles excluded, with reasons
(n=14)

Non-comparative records (n=2)

Transoral endoscopic thyroid surgeries (n=8)

Case reports (n=3)

Review (n=1)

Records after duplicates removed
(n=189)

Studies included in quantitative synthesis (meta-analysis)
(n=15)
